# Plastic pollution in the Amazon: The first comprehensive and structured scoping review

**DOI:** 10.1007/s13280-025-02245-2

**Published:** 2025-09-13

**Authors:** Jéssica Fernandes de Melo, Daniel Tregidgo, Anamelia Jesus, Jesem Douglas Yamall Orellana

**Affiliations:** 1 Leônidas and Maria Deane Institute, Oswaldo Cruz Foundation, Rua Teresina, 476. Adrianópolis, Manaus, AM 69057-070 Brazil; 2 Present Address: Santa Cruz State University, Campus Soane Nazaré de Andrade, Rod. Jorge Amado, Km 16 - Salobrinho, Ilhéus, BA 45662-900 Brazil; 3 Mamirauá Institute for Sustainable Development, Estrada do Bexiga, 2584, Fonte Boa, Tefé, AM 69553-225 Brazil

**Keywords:** Amazon basin, Food security, One health, Plastic waste

## Abstract

Plastic pollution is a global One Health crisis, yet research has focused largely on marine environments. The Amazon—the world’s largest drainage basin and second most plastic-polluted river—has received limited scientific attention. This scoping review is the first to apply a systematic protocol (PRISMA-ScR) to assess plastic contamination across Amazonian ecosystems. We reviewed 52 peer-reviewed studies reporting plastic litter and fragments in terrestrial and aquatic environments of the biome. Most evidence concerns microplastics in Brazilian sites along the main Amazon River, with a focus on fish. However, plastics were also found in sediments, plants, and diverse fauna including birds, reptiles, and mammals. No studies reported nanoplastics. The contamination of key food and water sources poses a major One Health risk for traditional populations. We identify urgent research gaps—especially in non-fish fauna, tributaries, and other Amazonian countries—and highlight the need for targeted mitigation through waste management and education.

**Supplementary Information:**

The online version contains supplementary material available at 10.1007/s13280-025-02245-2.

## Introduction

Globally, whether in the water, soil, or air, plastic is one of the most omnipresent contaminants in the environment. Its presence compromises ecosystem functions and poses serious health risks to wildlife and humans alike, highlighting plastic pollution as a One Health issue (MacLeod et al. 2021; Morrison et al. 2022). Plastic pollution affects the pillars of One Health, which are based on the interdependence of human, animal, and environmental health, emphasising that threats to any one pillar also threaten the others. For over half a century (Carpenter and Smith 1972; Carpenter et al. 1972), scientists have documented plastic litter (macroplastics) and later plastic fragments (meso-, micro- and nanoplastics, defined as particles > 5 mm and < 25 mm, < 5 mm, and < 1000 nm, respectively) build up in ecosystems across the planet (Barbosa et al. 2020; Napper and Thompson 2023). Much of the global public are now aware of how plastic litter can harm wildlife through entangling or ingestion, while scientific evidence of the numerous impacts (such as inhibiting feeding and fertility) of plastic fragments on marine, and less-so freshwater and terrestrial fauna, is increasingly accumulating (Zolotova et al. 2022).

Although the effects of plastic fragments on human health remain poorly understood, many chemicals associated with plastic are known to be hazardous and linked to a variety of diseases (Montenegro et al. 2020; Jones 2024). Microplastics have been detected in human tissues, including in the placenta, brain, breast milk, blood, semen, ovarian and even in the heart (Ragusa et al. 2021; Yang et al. 2023; Li et al. 2024; Lourenço et al. 2024; Montano et al. 2025). Microplastics can enter the human body through inhalation of air (Luo et al. 2024), but studies from modern food systems (HLPE 2017) conclude that the main route of entry to be through drinking water and seafood consumption (WWF 2019). However, in traditional food systems, where much of the food and drinking water is sourced from natural water bodies and forests, exposure risks remain understudied and may be significantly higher.

As the largest tropical rainforest and freshwater basin, the Amazon is also home to a megadiversity of fauna, and human populations that are heavily dependent on natural resources for subsistence, including thousands of traditional riverine and indigenous communities (Chowdhury et al. 2024; Orellana et al. 2025). Some Amazonian riverine communities exhibit among the highest per capita fish consumption in the world (240–293 kg/year; Tregidgo et al. 2023), exceeding even the Maldives—the world’s top fish-consuming nation (up to 185 kg; FAO 2014). Urban areas in the Brazilian Amazon also have high fish consumption, with some towns consuming four times the national average (Ferraz and Barthem 2016). This high dependency on fish, already a concern in the context of mercury exposure (Lopes et al. 2020), underscores the need to understand emerging contaminants such as Amazonian plastic pollution through a One Health lens—an integrated perspective that recognises the interconnections between human, animal, and environmental health (Wang et al. 2024). While the health impacts of mercury are well-documented in some Amazonian regions, the potential health outcomes of ingesting or inhaling plastic are largely neglected and unknown. Drinking water is also commonly obtained from natural water bodies in many riverine communities, often with little or no treatment (Gomes et al. 2022), presenting a significant potential source of plastic ingestion.

Amazonia has gained attention as a “new frontier on plastic waste”, with the Amazon River contributing significantly to oceanic plastic pollution, potentially accounting for 10% of marine plastic emissions (Lebreton et al. 2017; Giarrizzo et al. 2019). While oceans often take centre stage, the impacts on terrestrial and freshwater ecosystems are equally critical, particularly in the Amazon, where food and water insecurity, along with sanitation challenges, magnify the effects on local communities and biodiversity (Gomes et al. 2022; Albert et al. 2023; Chowdhury et al. 2024; IBGE 2024; Orellana et al. 2025).

The Amazon’s rivers are not only vital for subsistence but also serve as significant conduits for plastic waste, affecting aquatic fauna and contaminating essential food resources. This direct cycle of contamination—from environment to fauna to humans—demonstrates the One Health threat posed by plastic pollution in the Amazon and underscores its relevance (Fernández-Llamazares et al. 2020). This scoping literature review aims to explore and synthesise research on presence of plastic (macro-, meso-, micro-, and nanoplastic) pollution across the Amazon. It will assess existing research on plastic contamination within various environmental components—fauna, flora, sediment, and water—in the form of a scoping literature review, representing the first to apply a standardised systematic approach to assess Amazonian plastic pollution.

## Methods

A scope review was conducted following the Preferred Items for Systematic Reviews and Meta-Analyses extension for Scoping Reviews (PRISMA-ScR), crucial to ensure high-quality, sensitive, and transparent systematic literature reviews, or even to provide essential and reliable information for mapping and summarising the state of the art in a field related to certain scientific knowledge (Trico et al. 2018). The study protocol was planned to find original peer-reviewed articles (full paper or short communication) reporting plastic litter and plastic fragments in the biotic and abiotic components of terrestrial and aquatic environments of the Amazon biome, which encompasses the following countries: Brazil, Peru, Bolivia, Colombia, Ecuador, Venezuela, Guyana, French Guiana, and Suriname. The search took place from the year 2000, when the first original peer-reviewed articles involving the topic in the Amazon were published, until 21 April 2025, without language restrictions. Articles that were not conducted in the Amazon, that did not report the presence of plastic and its derivatives in the environment (i.e., soil, water, fauna, or flora) and ex situ experimental studies were excluded from the analyses. Web of Science, PubMed and Google Scholar were selected as the databases for the searches. The search key for the main database (Web of Science) was the following:

*(TS* = (plastic* OR microplastic*) AND TS = (amazon*)) AND (CU = *(BRAZIL or PERU or BOLIVIA or COLOMBIA or ECUADOR or VENEZUELA or GUYANA or FRENCH GUIANA or SURINAME)) AND (PY* = *(2000–2025)) AND (WC* = *(Environmental Sciences or Marine Freshwater Biology or Ecology or Zoology or Fisheries or Plant Sciences or Forestry or Multidisciplinary Sciences or Public Environmental Occupational Health or Biodiversity Conservation or Veterinary Sciences or Water Resources or Biology or Meteorology Atmospheric Sciences or Soil Science or Toxicology or Chemistry Multidisciplinary or Geography Physical)) NOT (TS* = *(plasticity)).*

The literature search was performed in all databases using the following combination of Boolean operators: “AND”, “OR” and “NOT”. All articles retrieved were collected using the Zotero software and organised into folders by database. After removing duplicates, articles were selected by reading the title and abstract, to exclude those that did not meet the aforementioned criteria. This screening was carried out by two authors, independently, to then review the inconsistencies found and resolve them by consensus.

After filtering the articles, the selected publications were read in full. The variables extracted from each article, when available, were: Title; Authors; Publication year; Sample year; Study location/area; Country; Language; Latitude; Longitude; Study focus (fauna, flora, sediment, and water); Collection method; Plastic detection method; Plastic shape; Plastic size; Category of plastic size; Probabilistic sample or not; Plastic colour; Reference.

*For studies focusing on fauna:* Taxon; Name; Number of species analysed; Number of species positive; Number of individuals analysed; Number of positive individuals; Type of interaction with plastic; Analysed body part; Average particle/individual; Consumption by the local population.

*For studies focusing on sediment, water and flora:* Plastic abundance; Density; Comparison.

**Fig. 1:**
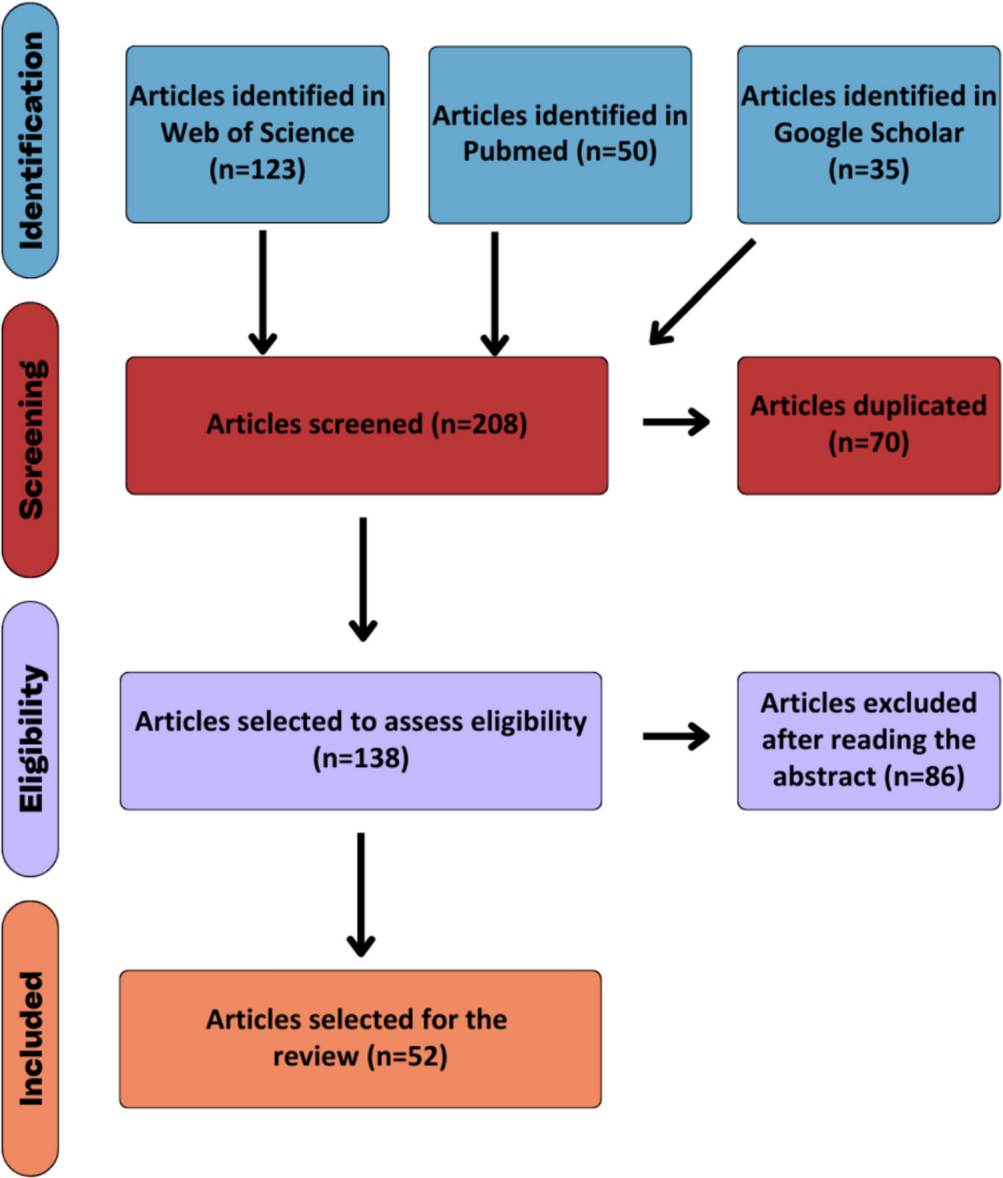
PRISMA flow chart showing the protocol followed to select the articles

A PRISMA flow chart (Fig. 1) was constructed to represent each stage of the literature search (Tricco et al. 2018). The study area for each article was defined based on the main river, bay or coast where data collection took place. The plastic size was divided into macro (> 25 mm), meso (5–25 mm), micro (< 5 mm) and nano (< 1000 nm) (Bilal and Iqbal 2020). Where the study methods were designed specifically to assess the presence of plastic particles in the environment the work was defined as “probabilistic”. Where it was not, for example where there was an opportunistic record of plastic during research with another objective, the article was defined as “non-probabilistic”. The consumption of the studied fauna by the local population was defined either by expert knowledge from authors based in the Amazon, or by consulting the relevant literature (e.g., Santos et al. 2009).

## Results

The searches in the three databases found a total of 208 articles. After removing duplicates, 138 articles remained to read the title and abstract for screening, when 86 articles were excluded based on the PRISMA methodology criteria. The 52 articles selected for the review are summarised in Table 1. Of these, 49 (94.2%) articles are in English, two (3.8%) in Portuguese and one (1.9%) in Spanish.

**Table 1:** Articles found in the systematic research containing information about plastic particles in the Amazon. *Animal consumption by local people

Authors	Publication year	Study location/area	Country	Language	Study focus	Animal local consumption*	Collection method	Plastic size	Probabilistic
Silva and Marmontel	2009	Solimões/Japurá River	Brazil	Portuguese	Fauna	Yes	Semi-structured interviews	macro	No
Guterres-Pazin et al.	2012	Solimões/Japurá River	Brazil	English	Fauna	Yes	Faecal samples and stomach contents of animals hunted or incidentally caught	macro	No
Lenoir et al.	2016	Sinnamary River basin	French Guiana	English	Fauna	NA	Active collection of animals	NA	Yes
Pegado et al.	2018	Amazon coast, between Amapá and Pará states	Brazil	English	Fauna	Yes	Collection of bycatch shrimp fishery	micro	No
Andrade et al.	2019	Xingu River	Brazil	English	Fauna	Yes	Active collection of animals	micro and meso	Yes
Martinelli Filho et al.	2019	Amazon coast, Pará state	Brazil	English	Sediment	NA	Sandy beach sediment samples	micro	Yes
Chota-Macuyama et al.	2020	Amazon River	Peru	Spanish	Fauna	Yes	Specimens obtained from local markets	micro	Yes
Gonçalves et al.	2020	Pará River	Brazil	English	Sediment	NA	Collection of litter	macro	Yes
Gerolin et al.	2020	Negro, Solimões and Amazon Rivers	Brazil	English	Sediment	NA	river bottom sediment samples	micro	Yes
Novaes et al.	2020	Marajó Bay, Pará	Brazil	English	Sediment	NA	Beach sediment samples	micro	Yes
Morais et al.	2020	Amazon coast, Pará state	Brazil	English	Fauna	Yes	Active collection of animals	micro and meso	Yes
Ribeiro-Brasil et al.	2020	Guamá and Acará-Capim Bay	Brazil	English	Fauna	Yes	Active collection of animals	micro and meso	Yes
Andrades et al.	2021	Marajó Bay, Pará	Brazil	English	Fauna	Yes	Opportunistic	macro	No
Pegado et al.	2021	Amazon coast, Maranhão state	Brazil	English	Fauna	Yes	Active collection of animals	micro	Yes
Fernandes et al.	2021	Amazon coast, Maranhão state	Brazil	English	Fauna	Yes	Active collection of animals by artisanal fisheries	meso	No
Lucas-Solis et al.	2021	Misahualli River basin	Ecuador	English	Sediment	NA	Beach sediment samples	micro	Yes
Lima et al.	2022	Maranhão Island	Brazil	English	Sediment	NA	Collection of debris	macro	Yes
Queiroz et al.	2022	Brazilian Amazon Continental Shelf (Maranhão to Amapá states)	Brazil	English	Water	NA	surface water samples	micro	Yes
Alfred et al.	2022	Guyana coast	Guyana	English	Fauna	Yes	Active collection of animals	micro and meso	Yes
Trindade et al.	2023	Xingu River	Brazil	English	Fauna	No	Active collection of animals	micro and meso	Yes
Oliveira et al.	2023	Itacoatiara, Amazonas state	Brazil	English	Sediment	NA	Areas used and not used for recreation	micro	Yes
Firmino et al.	2023	Negro River basin	Brazil	English	Fauna	NA	Active collection of animals	micro	Yes
Rosa et al.	2023	Guajará Bay, Acará River and Guamá River	Brazil	English	Water	NA	Visual observation of litter in transects	macro	Yes
Dantas Filho et al.	2023	Madeira River basin	Brazil	English	Water	NA	Surface water samples	micro	Yes
Rico et al.	2023	Belém River	Brazil	English	Water	NA	Surface water samples	micro	Yes
de Souza et al.	2023	Negro River	Brazil	Portuguese	Water	NA	Surface water samples	micro	Yes
Guimarães et al.	2023	Solimões River	Brazil	English	Fauna	Yes	Active collection of animals	micro	Yes
Rojas et al.	2023	Amazon River	Peru	English	Fauna	Yes	Specimens obtained from local markets	micro	Yes
Santos et al.	2023	Guajará Bay and Guamá River	Brazil	English	Water	NA	Surface water samples	micro and meso	Yes
da Costa et al.	2023	Machado River basin	Brazil	English	Fauna	Yes	Active collection of animals	micro	Yes
Correia et al.	2023	Xingu River basin and Caeté River	Brazil	English	Fauna	No	Active collection of animals	micro	Yes
Assis et al.	2023	Caeté River estuary	Brazil	English	Sediment	NA	Collection of litter	macro	Yes
Oliveira et al.	2023	Tapajós River	Brazil	English	Water	NA	Subsurface water samples	micro	Yes
Pantoja et al.	2024	Amazon coast, Pará state	Brazil	English	Fauna	Yes	Specimens obtained from local markets	micro	Yes
Azevedo et al.	2024	Solimões River	Brazil	English	Fauna	Yes	Specimens obtained from local markets	micro	Yes
Rodrigues et al.	2024	Caeté and Piaba River estuary	Brazil	English	Fauna	Yes	Specimens obtained from local markets	micro	Yes
Guimarães et al.	2024	Amazon River	Brazil	English	Flora	NA	Collection of biomass and residues associated with macrophyte banks	macro, meso and micro	Yes
Lopes et al.	2024	Amazon coast, Pará state	Brazil	English	Fauna	No	Collection of abandoned nests	macro and meso	Yes
Mendes et al.	2024	Amazon coast, Pará state	Brazil	English	Sediment	NA	Collection of sediments in tidal channels	micro	Yes
Monteiro et al.	2024	Amazon coast—Amapá, Pará and Maranhão states	Brazil	English	Fauna	No	Visual observation	macro	No
Rosa et al.	2024	Tamandaré and Tucunduba rivers, Pará state	Brazil	English	Water	NA	Visual observations of solid waste	macro	Yes
Guimarães et al.	2024	Amazon River, Pará state	Brazil	English	Fauna	Yes	Fish markets	micro	Yes
Rico et al.	2024	Amazon River mouth	Brazil	English	Flora	NA	Soil samples	micro	Yes
Alencastre-Santos et al.	2024	Altamira, Medicilândia, and Vitória do Xingu, Pará state	Brazil	English	Fauna	No	Collection of animals with mist nets	micro	Yes
Mendes et al.	2024	Ajuruteua Peninsula, Pará state	Brazil	English	Water	NA	Water samples collected in the subsurface layer of the water column	micro	Yes
Lima et al.	2024	Amanã Sustainable Development Reserve, Amazonas	Brazil	English	Fauna	No	Blubber samples	NA	Yes
Mendes et al.	2025	Atalaia Natural Monument, Pará state	Brazil	English	Fauna	No	Visual observation	macro	Yes
Souza-Ferreira et al.	2025	Gunma Ecological Park, Pará state	Brazil	English	fauna	No	Active search and manual collection of animals	micro	Yes
Morais et al.	2025	Salinópolis, Pará	Brazil	English	Fauna	No	Collection of animals from rocky outcrops	micro and meso	Yes
Santos et al.	2025	Marajó Bay, Pará	Brazil	English	Fauna	Yes	Collection of animals by both scientists and local community	micro and meso	Yes
Tuvikene et al.	2025	Negro and Solimões Rivers, Amazonas	Brazil	English	Fauna	Yes	Collection of animals with multimesh gillnets	micro	Yes
Carvalho et al.	2025	Maranhão Island	Brazil	English	Fauna	Yes	Collection of animals with a 35-mm mesh size net	micro	Yes

**Fig. 2:**
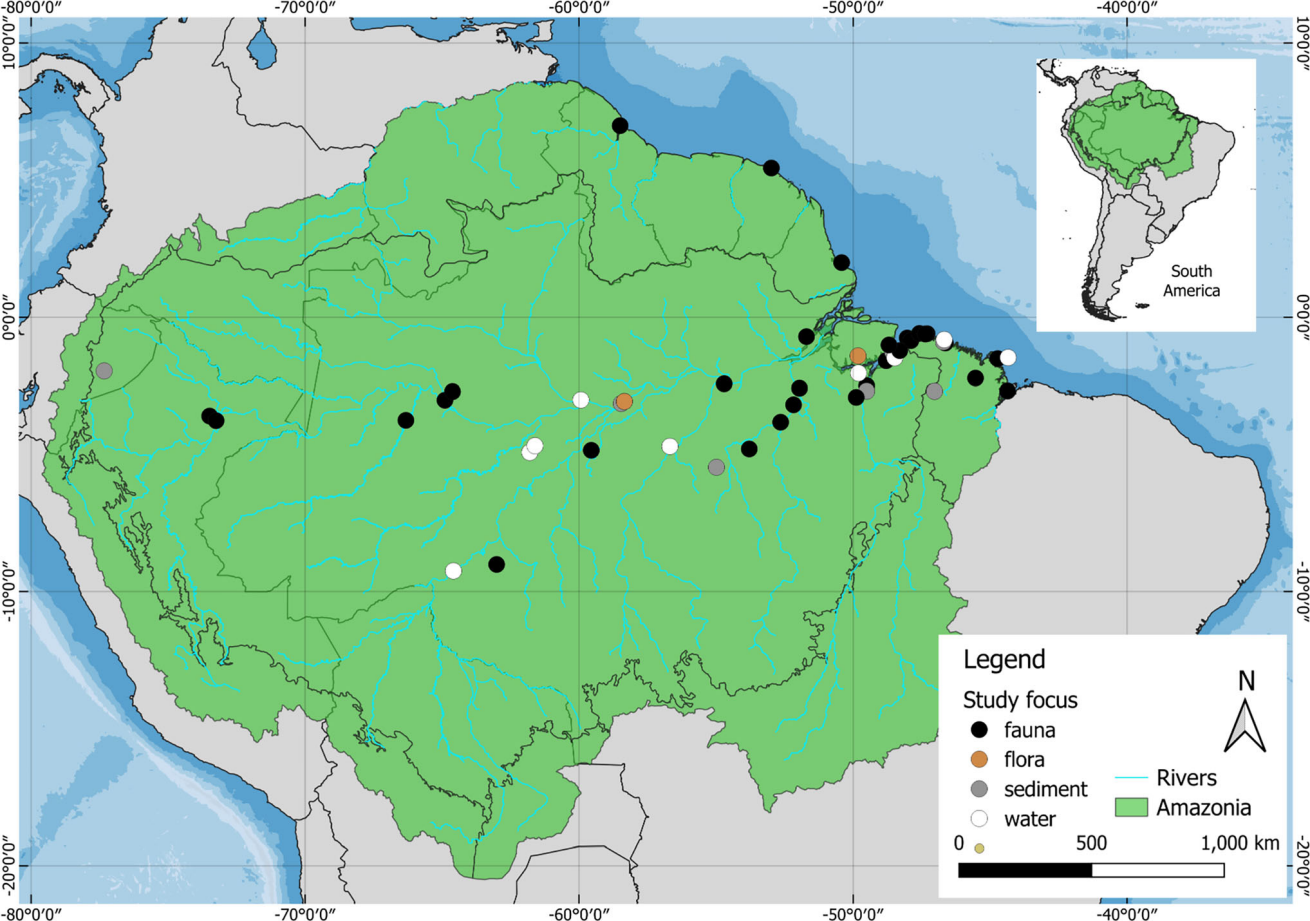
Geographical distribution of records of plastic litter and fragments in the Amazon biome

Regarding the study area of published works, only five were carried out outside Brazil—in Peru, French Guiana, Guyana and Ecuador. Most were undertaken in the central Amazon, roughly following the course of the River Amazon main channel until its mouth (Fig. 2). There is also a large concentration of studies in the eastern Brazilian state of Pará, located in the eastern/coastal Amazon. Other parts of the Amazon, such as the margins of the Amazon biome and the tributaries of the River Amazon, have practically no research on plastics pollution in the environment, as well as in indigenous lands.

**Fig. 3:**
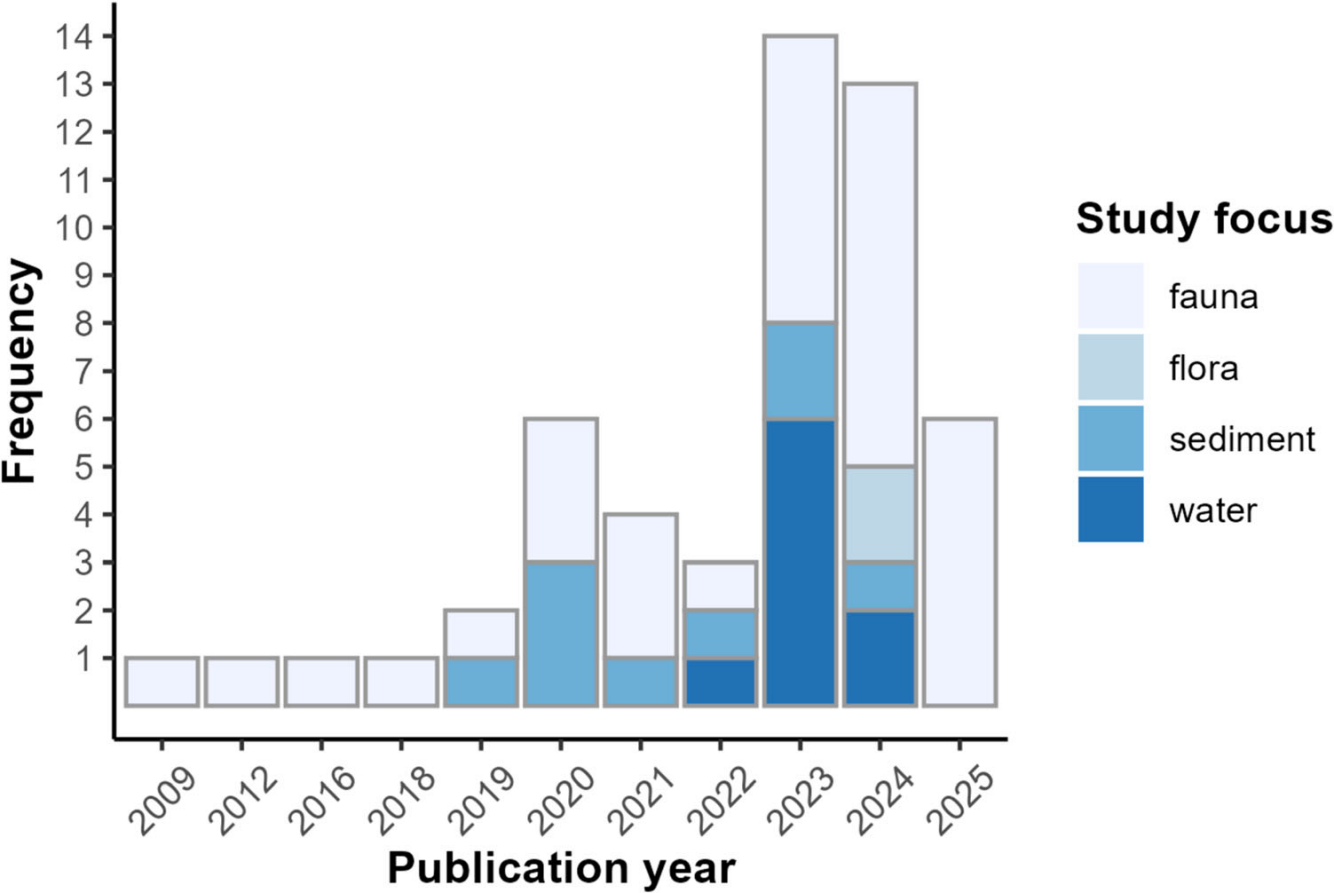
Absolute frequencies of articles published according to year and study focus—fauna, sediment or water, 2009–2025*. *For the year 2025, data collection was carried out until 21st April

The studies focused on fauna (*n* = 32, 61.5%), sediment (*n* = 9, 17.3%), water (*n* = 9, 17.3%) and two studies focused on flora (3.8%). The first article concerning plastic in wildlife in the Amazon was published in 2009 (Silva and Marmontel 2009), being a non-probabilistic report of the death of an Amazonian manatee (*Trichechus inunguis*), likely suffocated by a plastic bag in 2008. The next article on the topic was only published in 2012, another in 2016 and 2018 and two more in 2019. However, this subject only began gaining significant scientific attention in 2020, with a marked increase in published studies on the topic in subsequent years (Fig. 3). In other words, since the first important case, involving the death of a large mammal due to the presence of plastic in the environment, there was a gap of ten years for the topic to gain importance in research. Additionally, the presence of plastic particles in water samples from the Amazon was only published as of 2022.

**Fig. 4:**
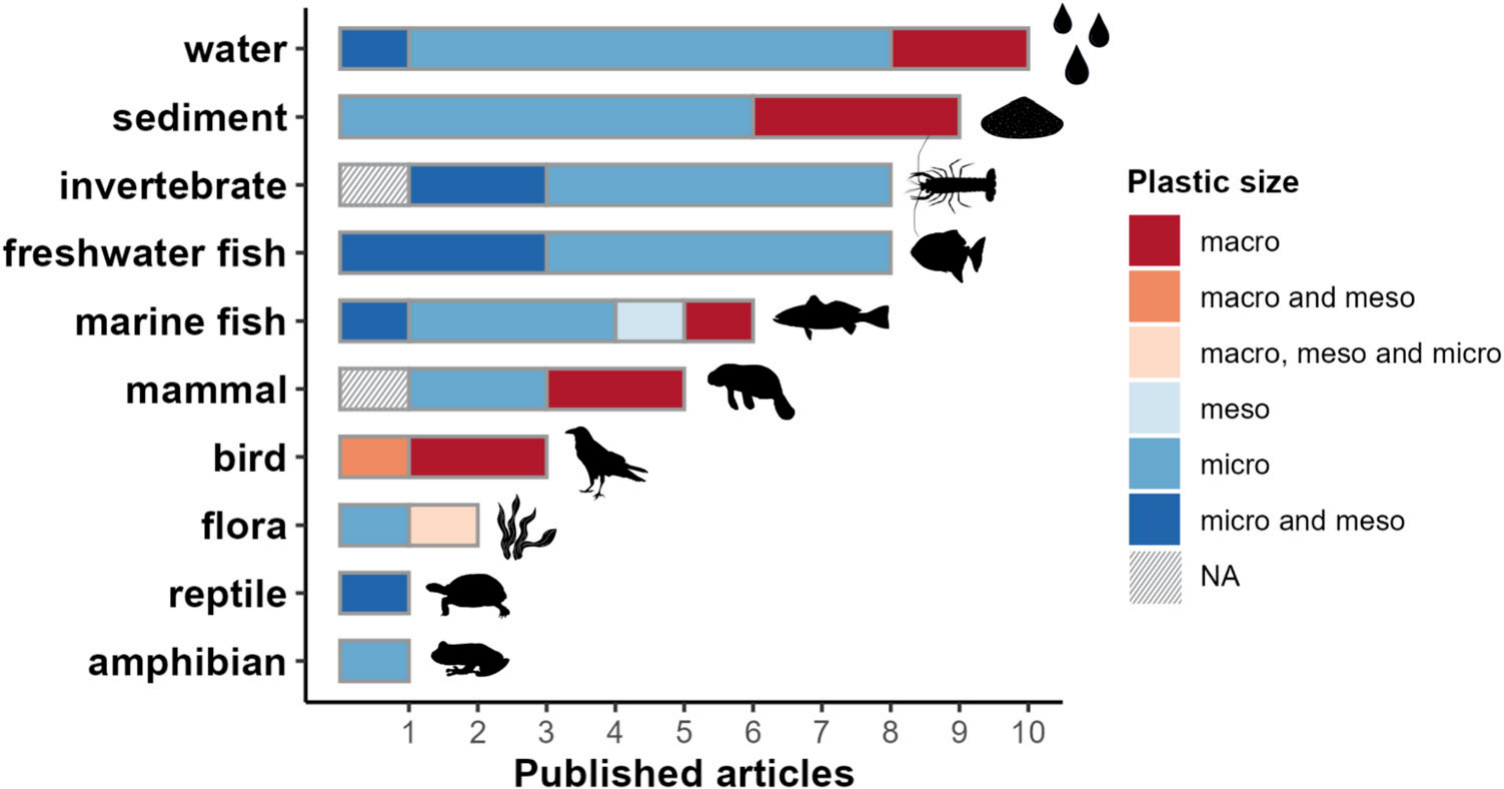
Absolute frequencies of published articles, according to study focus and plastic size

Most studies were focused on microplastics (*n* = 38, Fig. 4), while less found macroplastics (*n* = 12) and mesoplastics (*n* = 11), and none reported nanoplastics. Studies focused on fauna mostly investigated freshwater fish (*n* = 8) and invertebrates (*n* = 8), followed by marine fish (*n* = 6), mammals (*n* = 5), birds (*n* = 3), amphibian (*n* = 1) and reptile (*n* = 1). Studies focused on water found macro-, meso-, and microplastics. Macroplastics in water were detected through visual observation and included toys, straws, jerry cans, plastic bottles, plastic pieces, dishes and cups, labels/containers, plastic bags, container caps, bottle caps, utensils, garbage bags and pots. Fibres, fragments, and filaments were the most prevalent types of micro- and mesoplastics in water, primarily detected through Fourier transform infrared spectroscopy (FTIR). In sediment, microplastics were also commonly found, mainly represented by fibres and fragments, primarily identified through stereomicroscope use. In fauna, studies recorded micro- and mesoplastics (mainly fibres and fragments) through FTIR and stereoscopy. Macroplastics (such as plastic bags) were identified by direct observation. The characteristics of the plastic particles analysed (i.e. colour and type) are summarised in Appendix 10.1007/s13280-025-02245-2.

For most fauna studies, animals were captured or sampled in their natural habitat (*n* = 24; 75100%) by methods such as live capture and observational sampling. Other studies obtained animal samples from local markets or on farms (*n* = 6; 19%). Additionally, one study employed semi-structured questionnaires with artisanal fishermen to gather information (3%). Studies with birds showed plastic fragments associated with nests (*n* = 3, 9%). Domestic animals were excluded from the review if encountered in the search process.

All studies focusing on water and sediment were probabilistic, and only six studies (11%) on fauna were non-probabilistic—that is, the identification of plastic in samples was not the main objective of the study. The majority (66%) of animals researched are consumed by the local population (*n* = 21, Table 1), which further increases the relevance of such studies to human health. No studies were found with other classes of animals, and notably there was no research with humans.

Another interesting fact about the articles produced on plastic fragments found in the Amazon environment is regarding the authorship of the studies. In 22 (42.3%) of the 52 articles selected, the same individual appeared as a co-author in at least three or even 10 different articles. Between 2023 and 2025, 33 articles were selected and a group of five co-authors signed 9 (27.3%) of these.

## Discussion

This is the first review to systematically synthesise the existing research on environmental plastic (nano-, micro-, meso- and macroplastic) pollution in the Amazon, revealing widespread contamination throughout much of the geographical extent of the Amazonian biome, and in a large diversity of terrestrial and aquatic, abiotic and biotic specimens. This paper alerts to the extent and seriousness of the issue, emphasising the potential human health threat, as most contaminated specimens (water and fauna species) are commonly consumed by Amazonians. Although our review is largely limited to studies conducted in Brazil (90.4%), by including 52 articles—more than double or even triple the number captured in previous reviews (dos Santos Silva et al. 2024; Souza et al. 2023b; Morais et al. 2024)—it not only broadens the regional perspective on the issue but also provides an updated and reinforced understanding of the urgency of plastic pollution. We discuss the huge gaps in research and action necessary to understand and mitigate the issue.

### Amazonian plastics research is in its infancy

Silva and Marmotel (2009) published the first evidence of plastic contamination in the Amazonian environment in an opportunistic study of an Amazonian manatee. The first research specifically targeting plastic contamination was, however, only published in 2018 (Pegado et al. 2018), many decades after the establishment of marine plastic pollution literature (Carpenter and Smith 1972; Carpenter et al. 1972), demonstrating how Amazonian plastic pollution research is still in its relative infancy. More research is clearly needed, and once a critical mass of studies is available, this could also open the possibility to other kinds of systematic reviews (Page et al. 2021). Since these early papers, the Amazonian plastics pollution literature has grown in number, geographical extent, ecological realms, sample type and methodology, but is limited to just 52 original papers, even after a surge in publications in the past three years.

While anecdotal evidence of plastic contamination has existed for many years, shortly after the first targeted research evidencing plastic contamination in the Amazon, researchers labelled the biome “the new frontier for plastic pollution”. Three laboratories have identified the seriousness of plastic pollution in the Amazon, reviewing studies on plastic contamination and sources (dos Santos Silva et al. 2024; Souza et al. 2023b; Morais et al. 2024) in recent papers, with a focus specifically on microplastics. These reviews, respectively, identify 12 (Souza et al. 2023b) and 19 (dos Santos Silva et al. 2024; Morais et al. 2024) papers evidencing microplastics from field (ecosystem) studies, with Morais et al. 2024 presenting a further five experimental (laboratory) studies.

Utilising standardised scoping review methods and adhering to the steps outlined in the PRISMA protocol, such as delineating eligibility criteria—in this case, excluding experimental studies—and employing diverse databases with tailored search strategies, we compiled comprehensive evidence regarding plastic pollution in terms of both plastic litter (macroplastics) and fragments (micro- and meso- plastics) found in the environment in five Amazonian countries. Using this protocol, we identify nearly three times as many original field studies evidencing plastic pollution in the Amazon than in any previous review. This first systematic synthesis of published evidence of plastic pollution in the Amazon basin reveals a young but growing field of study. This upward trajectory is not only significant for the Amazon region itself but also holds broader implications, as a substantial proportion of plastic waste from Amazonian waterways ultimately finds its way into the world’s oceans. Estimates suggest that the Amazon contributes a staggering 10% of total oceanic plastic pollution (Giarrizzo et al. 2019).

Beyond thematic priorities, our review reveals a structural issue: a small group of researchers dominates the literature, with five co-authors responsible for over a quarter of articles published between 2023 and 2025. This points to a lack of diversity among research groups, which may limit both geographic coverage and methodological innovation. We highlight the need to foster new teams—particularly in underrepresented regions—to expand perspectives and approaches. This also reinforces the call for more rigorous systematic reviews, moving beyond scoping reviews that risk perpetuating existing biases.

### Sediment, water, flora and fauna samples from aquatic and terrestrial environments

The papers in this review identified plastic contamination associated with Amazonian water, sediment, flora, birds, frogs, freshwater fish, marine fish, both aquatic and terrestrial invertebrates, and both aquatic and terrestrial mammals.

Global plastic pollution research is biased towards aquatic and particularly marine environments (Kallenbach et al. 2022; Zolotova et al. 2022). Most Amazonian plastics studies were from freshwater environments, but several terrestrial studies were also identified. The fact that microplastics have now been identified in Amazonian ants (Lenoir et al. 2016), tree frogs (Souza-Ferreira et al. 2025) and bats (Correia et al. 2023) suggests that other terrestrial animals that feed on aquatic animals, or simply consume water from rivers, may also be contaminated with plastics, which presents a health risk for both these animals and their predators. Three recent studies found in our search present the first published evidence of plastics associated with Amazonian birds (Lopes et al. 2024; Monteiro et al. 2024; Mendes et al. 2025), in the form of macroplastics in nests. Research from other regions has shown nestlings entangled in similar plastic strings (Janic et al. 2023), suggesting a risk to Amazonian species too. It is important to make efforts to understand the risks of plastic particles contaminating Amazonian terrestrial fauna, especially animals that are part of the diet of riverine and indigenous people in the form of wildmeat.

Regarding sediments contaminated by plastic, Gonçalves et al. (2020) showed that habitats with vegetation are acting as a litter sink for macroplastics, more than habitats free of vegetation. This is worrying for aquatic fauna, in the context of studies showing macroplastics resulting in the death of manatees and fish (Silva and Marmontel et al. 2009 and Andrades et al. 2021). In addition to these, other herbivorous animals such as turtles, capybaras, tapirs, peccaries, pacas, waterbirds, may be eating the plastic that is lodged among the vegetation. Specifically, macroplastics may also present a greater risk to fauna.

### Nano-, micro-, meso- and macroplastic fragment sizes

We present studies identifying environmental contamination by plastic litter (macroplastics) and plastic fragments (micro- and mesoplastics), although we found no evidence of nanoplastic contamination. Large pieces of plastic litter (macroplastics) visually dominate some Amazonian streams (e.g., Giarrizzo et al. 2019; Lima et al. 2022) and are responsible for injury or death of wildlife through entanglement and ingestion. For example, the first published evidence of plastic contamination in the natural environment in the Amazon was a plastic bag in a manatee, which authors presume to have been the cause of death. Guterres-Pazin et al. (2012) also found macroplastic (5 × 2 cm) in stomach content of a manatee, and Andrades et al. (2021) reported a dead fish suffocated by a plastic bag in the Amazon estuary. Therefore, macroplastics can pose an imminent danger to aquatic and terrestrial wildlife, which could also include nestlings tangling in the plastic fibres that make up their nests. However, the dangers of plastics that are not visible to the naked eye also need attention.

Three quarters of the studies in this review identify microplastics (73%), which follows the global research focus on this specific fragment size, including existing Amazonian literature reviews (dos Santos Silva et al. 2024; Souza et al. 2023b; Morais et al. 2024). Integrating research across all plastic fragment sizes is essential for developing comprehensive strategies to address plastic pollution effectively. The lack of research on nanoplastics in the Amazon is notable, especially as they are thought to be more widespread, and potentially more dangerous to people and wildlife due to their ability to penetrate living cells (Sharma et al. 2023). This could partially be due to the difficulty of detecting them, for example due to the current lack of laboratory equipment able to detect nanoplastics in the nine Amazonian countries (to the best of our knowledge).

### The spatial extent of plastic contamination in the Amazon

Figure 2 shows that plastic contamination has been found from the western to the eastern Amazon, and that most evidence roughly follows the main Amazon River channel, with considerably less evidence up the tributaries. This is likely to be due to sampling effort and coincides with detailed maps of biodiversity sample effort, which predictably shows more sampling effort in more accessible locations (Santos et al. 2015). More studies were found in the eastern amazon, i.e. downstream, which will receive plastic from upstream, i.e. the western Amazon and Andes mountains. It may be seen as surprising that almost all (90%) of the studies are in one country (Brazil), with no studies in four of the nine Amazonian countries. There is therefore a clear need to expand plastic pollution research to these understudied areas of the Amazon.

### Human health risk

Plastics can harm or be fatal to fauna through various means, including entanglement, interrupting nutrient absorption, causing reproductive problems, hindered growth and survival of young animals (Susanti et al. 2020). Plastic contamination in ecosystems not only threatens biodiversity but can also have cascading effects on food security, water quality, and human well-being. Yet, it is generally accepted that we do not understand the impacts of plastic contamination on human health. Research now shows plastic fragments in various human tissues (Ragusa et al. 2021; Yang et al. 2023; Amato-Lourenço et al. 2024; Montano et al. 2025), and we know that many of the chemicals associated with plastic are hazardous (Jones 2024). Moreover, recent landmark (albeit non-causal) research shows strong evidence that plastic contamination could be contributing to a large list of human health problems (Marfella et al. 2024).

Most of the studies identified in this review show plastic contamination in specimens, which are consumed by local human populations, who are highly dependent on natural water bodies for drinking water (Gomes et al. 2022) and local fish and wildmeat for food and nutritional security (Dufour et al. 2016; Tregidgo et al. 2023), particularly rural traditional populations. Most of the contaminated fish species identified in this review are known to be important food species. While plastic detected in the bodies of the studied terrestrial wildlife does not represent a direct threat to humans, as bats and the studied frog species are not known to be eaten in the Amazon, evidence of plastic contamination in terrestrial fauna suggests that it could be present in other terrestrial species that are commonly consumed by traditional populations and essential for adequate nutrition of the rural communities (Torres et al. 2022). The presence of plastic in aquatic non-fish fauna is also worrying, including manatee (Guterres-Pazin et al. 2012) and turtles (Santos et al. 2025), which are consumed by local people. This evidence in addition to ample evidence of plastic contamination among diverse marine wildlife taxa (Zantis et al. 2021) suggests that plastics may be omnipresent in Amazonian aquatic fauna.

Except for the studies describing macroplastics in a manatee (Silva and Marmontel 2009), a fish (Andrades et al. 2021) and bird nests (Lopes et al. 2024; Monteiro et al. 2024; Mendes et al. 2025), most of the evidence of plastic in fauna presented in this review was shown as fragments found in the digestive systems of the studied animals. While worrying, this does not mean that humans will consume this plastic, as digestive systems are rarely eaten, unlike many shellfish, which is evidenced to be the main source of human plastic consumption from food in some modern food systems from non-Amazonian populations (WWF 2019; Senathirajah et al. 2021). In Amazon populations, whole digestive systems are sometimes consumed in specific situations whereby animals such as prawns and small fish are eaten whole, or in local dishes (commonly with turtles and the *Arapaima gigas* fish) where parts of the digestive system are eaten (usually the intestines are cut open and thoroughly cleaned prior to cooking which likely significantly reduces the risk of plastic ingestion by human consumers). We did not find any study that analyses plastic content of muscle (meat) tissue in neither fish nor wildmeat, which means that the role of Amazonian foods in human plastic ingestion remains poorly understood.

### Future research and policy implications

This review suggests that plastic residues may be omnipresent in the waters, sediments, macrophytes, and aquatic and terrestrial fauna through the Amazon. We identify two areas of immediate research priority to understand the extent of the One Health challenge.

Firstly, are plastic residues found in non-aquatic wildlife? Logically, we might expect that plastic residues with an aquatic origin may be omnipresent among any wildlife with an aquatic or semi-aquatic life, including caiman, river dolphins, turtles and aquatic birds. There is evidence of plastic particles in terrestrial mammals (Ayala et al. 2023) from all over the Americas, and we have found evidence of plastic in the bodies of terrestrial ants and bats in the Amazon (Lenoir et al. 2016 and Correia et al. 2023), as well as in the nests of several bird species. Given the vast extent of Amazonian floodplain systems (Fleischmann et al. 2022), we might also expect some level of contamination through the increased interaction with plastic residues derived from floodwaters, both during the floods, and deposited on land once the flood water recedes.

Secondly, are humans in the Amazon ingesting plastic residues? Our review shows that some of the fish species that are consumed by Amazonians contain plastic residues in the digestive tract. While stomachs and intestines of fish (namely *Arapaima gigas*) and other aquatic wildlife (namely river turtles) are sometimes consumed in local dishes, the muscle tissue is by far the most consumed body part. Hence, it is currently not evidenced if local people from the Amazon are eating plastic, as there is no evidence of plastic residues in muscle (meat). It has been estimated that Australians may be eating approximately 5 g (equivalent to a credit card) of plastic per day, although most of this comes from drinking water, followed by shellfish, in which the digestive system is consumed (Senathirajah et al. 2021; WWF 2019).

Rainwater and groundwater are the main drinking water sources in riverine communities in flooded and unflooded areas, respectively, although drinking river water remains common (Gomes et al. 2022). Research on drinking water is needed to understand the extent of plastic ingestion. In terms of fish, and potentially wildmeat, it is important to understand whether nano-/microplastics are making their way into muscle tissue, which is being consumed by humans, as has been observed in marine fish (Akhbarizadeh et al. 2018; Barboza et al. 2020).

Microplastics are an artificial categorisation of plastic contaminants and are arguably less important than smaller nanoplastics in regard to contamination of edible muscle tissue from wild fish and wild meat by local communities. Through investment in appropriate equipment or partnerships, future plastics research must take all plastics contaminant sizes into account, including nanoplastics.

Given the extent of the plastic waste issue that this review identifies, we recommend public policies that promote plastic reuse (circular economy), reduce single-use plastics (e.g., bags, food and beverage packaging) and improve solid waste management, especially in areas lacking infrastructure like riverside and Indigenous communities. Artisanal fishing also contributes to pollution through discarded nets, lines and Styrofoam. Promising innovations like microbial plastic degradation and bioplastic production (da Silva Pereira et al. 2021; Roman et al. 2024) should also be explored in local communities, potentially supporting both environmental goals and the bioeconomy. Tackling the issue requires intersectoral collaboration among governments, companies, schools and civil society, with awareness initiatives—like a “Plastic-Free Day”—and targeted campaigns in schools, workplaces, transit hubs and public spaces to reduce everyday plastic use. Applying a One Health lens to Amazonian plastic pollution bridges environmental and public health while making the issue tangible for local communities—by revealing how plastic waste in rivers ends up in the fish they prize as food and cultural staples.

## Conclusions

This first systematic synthesis of the literature regarding plastic contamination in the Amazon presents a highly worrying scenario, supporting the previous label as “the new frontier for plastic pollution”. Evidenced in 52 scientific papers, plastic waste was detected from east to west of the immense Amazonian biome (twice the size of India), in terrestrial and aquatic systems, and in water, sediment, plants and fauna. While the evidence suggests that plastic is omnipresent, research is clearly in its infancy, and more research is needed in underrepresented portions of the Amazonian, in more non-fish fauna, and in regard to plastics of sizes other than microplastics (namely nanoplastics). This One Health approach could help us to understand the interconnected risks to the health of Amazonian water bodies and fauna, and the human population that depends on them for their Water Security and Food and Nutritional Security. We, however, argue that the evidence presented in this review is more than sufficient to justify immediate policy implementation throughout the Amazon to reduce plastic reaching the natural environment.

## Supplementary Information

Below is the link to the electronic supplementary material.

Supplementary file1 (XLSX 45 kb)

## Data Availability

The dataset supporting the conclusions of this article are included within 10.1007/s13280-025-02245-2.
